# Metagenomic Sequencing of Prokaryotic Microbiota from Tropical Surface Seawater

**DOI:** 10.1128/genomeA.00540-13

**Published:** 2013-08-15

**Authors:** Xin Yue Chan, Ramitha Arumugam, Siew Woh Choo, Wai Fong Yin, Kok Gan Chan

**Affiliations:** Division of Genetics and Molecular Biology, Institute of Biological Sciences, Faculty of Science, University of Malaya, Kuala Lumpur, Malaysiaa; Research and Training Unit, Faculty of Dentistry, University of Malaya, Kuala Lumpur, Malaysiab

## Abstract

Tropical seawater harbors a rich diversity of microorganisms as a result of its nutrient-rich environment, constant supply of sufficient sunlight, and warm climate. In this report, we present the complexity of the microbial diversity of the surface seawater of the Georgetown coast as determined using next-generation sequencing technology.

## GENOME ANNOUNCEMENT

Seawater harbors a great diversity of living organisms, ranging from microscopic bacteria, *Archaea*, fungi, and *Protista* to macroscopic crustacean and fishes, each playing a major role in the biogeochemical cycle (1, 2). South Channel, located at the tropical coast at the northern part of the Malacca Straits, Malaysia, is believed to harbor great oceanic biodiversity due to the warm seawater and consistent exposure to sunlight. According to several studies, the microbial diversity increases as one gets closer to the coastal area (3). Therefore, this study aims to investigate the marine microbial diversity of the surface seawater of the Georgetown coastal area.

A sterile amble bottle was used to collect 1 liter of water from the sea surface of the Georgetown coast (05°25.587′N, 100°19.591′E), Penang, Malaysia, and the water was filtered through a filter membrane (pore size of 0.22 µm) (Sartorius, Germany). Metagenomic DNA was extracted and purified with a modified cetyltrimethylammonium bromide (CTAB) DNA extraction method (4, 5). The purified DNA was processed for sequencing-library preparation with the TruSeq DNA sample preparation kit (Illumina) prior to shotgun sequencing with MiSeq (Illumina). A total of 6,701,060 raw reads were channeled to the CLC Genomic Workbench 5.1 (CLC bio, Denmark) for trimming and *de novo* assembly (6). Assembled sequences were compared against the Cyberinfrastructure for Advanced Microbial Ecology Research and Analysis (CAMERA) for microbial diversity, and its outputs were imported into MEGAN4 software (version 4.64.2) for classifications (7, 8). Microorganism abundance was assessed by counting the number of reads.

In terms of the kinds of organisms present, the majority were members of the *Eubacteria* and *Archaea*, making up 75% of the total organism presence. A total of 31 known bacterial phyla, together with a significant number of unclassified bacteria, were reported from this sample, suggesting rich bacterial diversity at the seawater surface of the Georgetown coast. Among the *Eubacteria*, the *Proteobacteria* (72%) make up the most abundance phylum, followed by the *Bacteroidetes* (9.4%), *Firmicutes* (5.1%), *Actinobacteria* (3.0%), *Verrucomicrobia* (1.8%), and *Cyanobacteria* (1.8%). On the other hand, the major phylum of the *Archaea* found in this sample was *Euryarchaeota*, contributing 65.2% of the *Archaea* diversity. Members of the *Thaumarchaeota* make up 17.4% and members of the *Crenarchaeota* make up 9.6% of the total *Archaea* presence in this sample, making these 2 of the most abundant phyla of *Archaea* found.

In this study, we report the rich microbial diversity found at the sea surface of the tropical Georgetown coast. The benefits of this great microbial diversity of the tropical marine waters, along with the diverse microbial gene activities, might be the key to enhancing the bioremediation dynamics of the polluted seaside.

### Nucleotide sequence accession number.

The DNA sequences from this metagenomic project were deposited at DDBJ/EMBL/GenBank under the accession no. SRP014764.

## References

[1] Whitman WB Coleman DC Wiebe WJ 1998 Prokaryotes: the unseen majority. Proc. Natl. Acad. Sci. U. S. A. 95:6578–6583 9618454 10.1073/pnas.95.12.6578 PMC33863

[2] Gutierrez T 2011 Identifying polycyclic aromatic hydrocarbon-degrading bacteria in oil-contaminated surface waters at Deepwater Horizon by cultivation, stable isotope probing and pyrosequencing. Rev. Environ. Sci. Biotechnol. 10:301–305

[3] Azam F Fenchel T Field J Gray J Meyer-Reil L Thingstad F 1983 The ecological role of water-column microbes in the sea. Mar. Ecol. Prog. Ser. Oldendorf 10:257–263

[4] Zhou J Bruns MA Tiedje JM 1996 DNA recovery from soils of diverse composition. Appl. Environ. Microbiol. 62:316–322 8593035 10.1128/aem.62.2.316-322.1996 PMC167800

[5] Murray MG Thompson WF 1980 Rapid isolation of high molecular weight plant DNA. Nucleic Acids Res. 8:4321–4325 7433111 10.1093/nar/8.19.4321 PMC324241

[6] Chan XY Chua KH Puthucheary SD Yin W-F Chan K-G 2012 Draft genome sequence of an *Aeromonas* sp. strain 159 clinical isolate that shows quorum-sensing activity. J. Bacteriol. 194:6350. 10.1128/JB.01642-12 23105081 PMC3486352

[7] Seshadri R Kravitz SA Smarr L Gilna P Frazier M 2007 CAMERA: a community resource for metagenomics. PLoS Biol. 5:e75. 10.1371/journal.pbio.0050075 17355175 PMC1821059

[8] Huson DH Mitra S Ruscheweyh HJ Weber N Schuster SC 2011 Integrative analysis of environmental sequences using MEGAN4. Genome Res. 21:1552–1560 21690186 10.1101/gr.120618.111 PMC3166839

